# Analysis of the association between Fc receptor family gene polymorphisms and ocular Behçet’s disease in Han Chinese

**DOI:** 10.1038/s41598-018-23222-8

**Published:** 2018-03-19

**Authors:** Donglei Zhang, Jieying Qin, Lin Li, Guannan Su, Guo Huang, Qingfeng Cao, Aize Kijlstra, Peizeng Yang

**Affiliations:** 1 The First Affiliated Hospital of Chongqing Medical University, Chongqing Key Laboratory of Ophthalmology and Chongqing Eye Institute, Chongqing, P. R. China; 2 First Hospital of Shanxi Medical University, Taiyuan, Shanxi P. R. China; 3 University Eye Clinic Maastricht, Maastricht, The Netherlands

## Abstract

Fc receptors are known to have a pivotal role in the initiation and regulation of many immunological and inflammatory processes. This study aimed to investigate the association of Fc receptor family gene polymorphisms with ocular Behçet’s disease (BD) in Han Chinese. A two stage case–control study was performed in 1022 BD cases and 1803 healthy controls. Twenty-three SNPs were genotyped using the MassARRAY system (Sequenom), TaqMan SNP Genotyping Assay and polymerase chain reaction-restriction fragment length polymorphism (PCR-RFLP) method. The expression of FCGR3A was examined by real-time PCR and cytokine production was measured by enzyme linked immunosorbent assay (ELISA). A significantly higher frequency of the FCGR3A/rs428888 CT genotype (Pc = 1.96 × 10^−7^, OR = 1.897) and a lower frequencies of CC genotype and C allele (Pc = 1.96 × 10^−7^, OR = 0.527; Pc = 7.22 × 10^−7^, OR = 0.554 respectively) were found in ocular BD as compared with controls. Functional experiments showed an increased FCGR3A expression (P = 0.005) and increased cytokine protein expressions of MCP-1, IL-1β and TNF-α by LPS stimulated PBMCs in CT carriers of FCGR3A rs428888 compared to CC carriers (P = 0.034; P = 0.025; P = 0.04; respectively). Our findings demonstrate that FCGR3A/rs428888 confers genetic susceptibility for ocular BD in Han Chinese.

## Introduction

Behçet’s disease (BD) is a systemic autoinflammatory disease featuring recurrent oral aphthae, relapsing bilateral uveitis, typical skin lesions and genital ulcers. However, many other organs including the vascular, neurological and musculoskeletal systems can also be affected^1–3^. It is primarily prevalent in populations from Asia and the Middle East^4^. To date, the exact pathogenesis of BD remains unknown, although genetic factors coupled with a triggering event may play a crucial role in its development^5–8^.

BD is generally regarded as a T cell mediated disorder^9–12^. Recent studies have however suggested an additive role for B cells in its pathogenesis^13–15^. As receptors for the Fc domain of immunoglobulins (Ig), Fc receptors (FcRs) are extensively distributed on various types of immune cells, and are involved in a variety of innate and adaptive immune responses. They promote the activation of B cells and mediate the specific recognition of antigens by leucocytes^16^. The Fc receptor family has a large number of members with different structures and functions, which are composed of six main subfamilies, according to their binding of specific immunoglobulin isotypes: FcA receptors (IgA), FcG receptors (IgG), FcD receptors (IgD), FcE receptors (IgE), FcM receptors (IgM) and FcR-like receptors (FcRLs)^17,18^.

Fc receptors are known to have a pivotal role in the initiation and regulation of many immunological and inflammatory processes, and gene polymorphisms identified so far, contribute to the development of a range of chronic inflammatory and autoimmune diseases^19–27^. Previous studies showed that FcAR was associated with the genetic predisposition to granulomatosis with polyangiitis (GPA), and susceptibility to a serious renal disorder^19^. FCGRs polymorphisms have been shown to play a role in the development of SLE^20^ and FCGR2 was reported to be associated with immune mediated diseases like type I diabetes, celiac disease (CD) and Kawasaki disease^21,22^. FCRL3 variants can confer risk to multiple sclerosis (MS) and RA^23,24^, and FCRL4 is associated with ankylosing spondylitis (AS) in Chinese Han^25^. The association between genetic polymorphisms of Fc receptors with BD has been tested in relatively small cohorts of BD patients and did not encompass the complete family of Fc receptor genes^26,27^. We therefore decided to expand these studies using a complete set of currently known Fc receptor gene polymorphisms in a large cohort of Chinese Han ocular BD patients.

## Results

### Features of recruited cases

The demographic characteristics and clinical features of the 1022 recruited ocular BD patients and 1803 controls are shown in Table 1. All cases had uveitis. The mean age of the BD patients was 34.1 ± 8.4 years (range, 9–61 years), and included 861 males (84.2%) and 161 females (15.8%). Twenty-three SNPs of various Fc receptor genes were genotyped successfully and did not deviate from the Hardy-Weinberg equilibrium in healthy controls.

**Table 1:** Clinical Features of ocular BD patients.

Clinical Features	Total	%
**BD Patients**		
Mean age ± SD	34.1 ± 8.4	
Female	161	15.8
Male	861	84.2
Uveitis	1022	100
Oral ulcer	967	94.6
Skin lesions	748	73.2
Genital ulcer	575	56.3
Pathergy reaction	214	20.9
Hypopyon	187	18.3
Arthritis	184	18.0
Relapsing vasculitis	32	3.1
Nervous system abnormalities	22	2.1
Gastrointestinal tract lesions	10	0.9
**Controls**		
Mean age ± SD	38.0 ± 11.2	
Female	649	36.0
Male	1154	64.0

SD = standard deviation.

### Genotyping results in the first phase study

Polymorphisms of Fc receptor genes were investigated in 449 BD cases and 658 healthy controls for the first-phase study. A decreased frequency of the FCGR3A/rs428888 CC genotype was found in BD (Pc = 2.02 × 10^−2^, OR = 0.551) (Table 2). The frequency of the FCGR3A/rs428888 CT genotype in cases was significantly higher compared to controls (Pc = 2.02 × 10^−2^, OR = 1.814) (Table 2). No statistically significant association was found between the other 22 SNPs tested and ocular BD (Supplementary Table S1). Linkage disequilibrium(LD) was estimated for the five SNPs of FCGR3A using our own data, and showed that there was not a strong LD between the tested SNPs (Table S2, Fig. S1).

**Table 2:** Polymorphisms of *FCGR3A/rs428888* in ocular Behçet’s Disease.

SNP	Stage	Genotype/Allele	BD (freq.)		Control (freq.)		*P* Value	Pc Value	OR (95% CI)
rs428888(*FCGR3A*)	First	CC	363	(0.808)	582	(0.884)	4.40E-04	2.02 E-02	0.551 (0.394–0.771)
		CT	86	(0.192)	76	(0.116)	4.40E-04	2.02 E-02	1.814 (1.297–2.537)
		C	812	(0.904)	1240	(0.942)	7.44E–04	3.42E-02	0.579 (0.420–0.798)
		T	86	(0.096)	76	(0.058)	7.44E–04	3.42E-02	1.728 (1.254–2.382)
	Replication	CC	467	(0.815)	1025	(0.895)	3.54E-06	1.63E-04	0.516 (0.289–0.685)
		CT	106	(0.185)	120	(0.105)	3.54E-06	1.63E-04	1.939 (1.461–2.574)
		C	1040	(0.908)	2170	(0.947)	7.82E-06	3.60E-04	0.543 (0.414–0.712)
		T	106	(0.092)	120	(0.053)	7.82E-06	3.60E-04	1.843 (1.405–2.418)
	Combined	CC	830	(0.812)	1607	(0.891)	4.26E-09	1.96E–07	0.527 (0.425–0.654)
		CT	192	(0.188)	196	(0.109)	4.26E-09	1.96E–07	1.897(1.528–2.354)
		C	1852	(0.906)	3410	(0.945)	1.57E–08	7.22E–07	0.554 (0.451–0.682)
		T	192	(0.094)	196	(0.055)	1.57E–08	7.22E–07	1.804 (1.467–2.218)

SNP, single nucleotide polymorphism; Pc, Bonferroni corrected p value; OR, odds ratio; 95% CI, 95% confidence interval.

### Genotyping results in the second phase study and combined study

To validate the significant association of the FCGR3A gene polymorphism with BD found in the first phase study, another set of 573 BD cases and 1145 healthy controls were evaluated in a second phase test. The results confirmed the lower frequencies of the FCGR3A/rs428888 CC genotype and C allele in BD (Pc = 1.63 × 10^−4^, OR = 0.516; Pc = 3.60 × 10^−4^, OR = 0.543 respectively) and the higher frequency of the CT genotype (Pc = 1.63 × 10^−4^, OR = 1.939) (Table 2). The combined data of the two studies showed a stronger significant association of rs428888 in FCGR3A with BD. (CC genotype: Pc = 1.96 × 10^−7^, OR = 0.527; C allele: Pc = 7.22 × 10^−7^, OR = 0.554; CT genotype: Pc = 1.96 × 10^−7^; OR = 1.897 respectively). (Table 2)

### mRNA expression and downstream inflammatory factors

**Figure 1:**
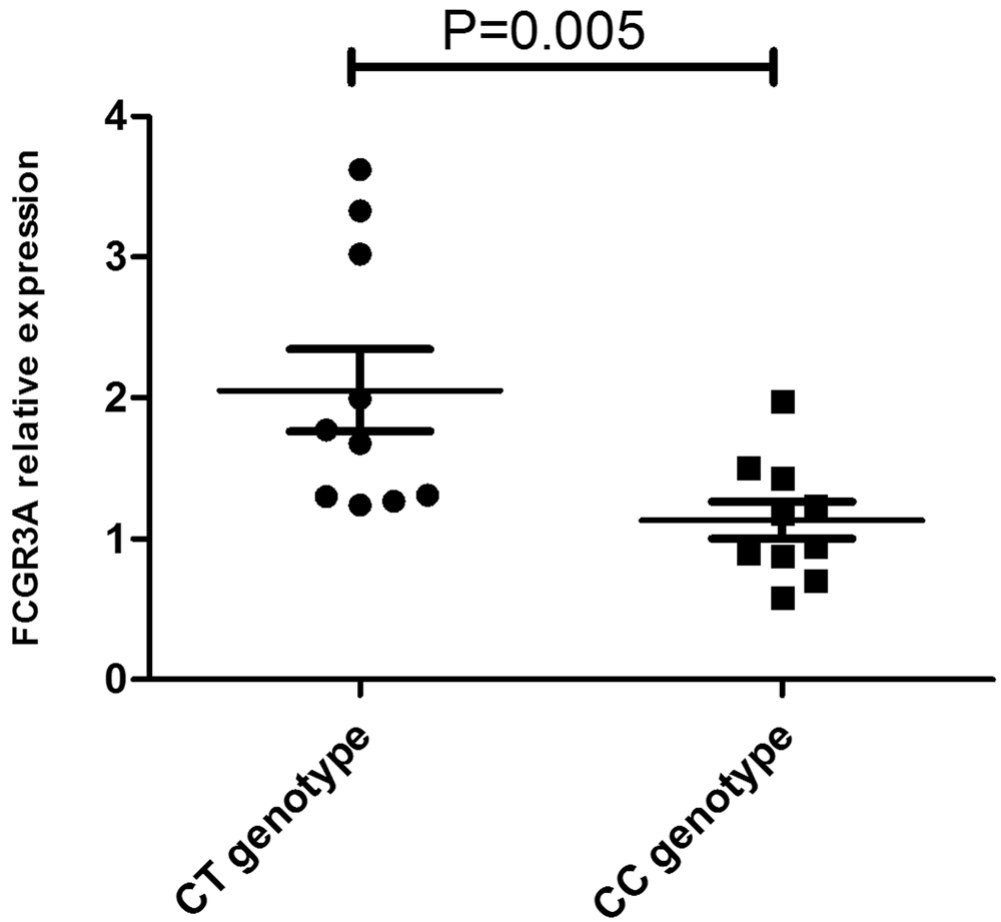
The influence of various rs428888 genotypes on FCGR3A expression The mRNA expression of FCGR3A in unstimulated PBMCs from healthy individuals with diverse genotypes of rs428888. (CT: n = 10, CC: n = 10); (male: n = 15, female: n = 5).

**Figure 2:**
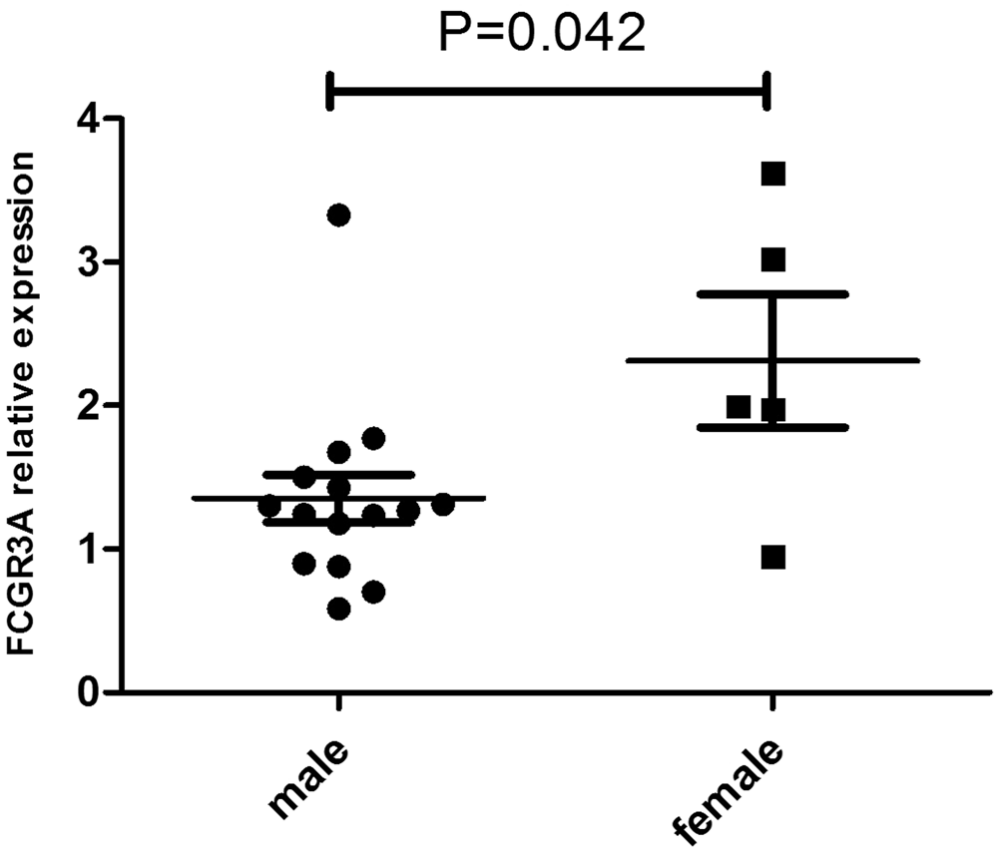
The influence of gender on FCGR3A expression The mRNA expression of FCGR3A in unstimulated PBMCs from rs428888 genotyped healthy individuals with a different gender. (male: n = 15 (CT: n = 7; CC: n = 8), female: n = 5 (CT: n = 3; CC: n = 2).

In view of the results shown above, we tested the biological function of the various genetic variants of FCGR3A. This was done by measuring the expression of FCGR3A in PBMCs obtained from healthy genotyped controls since the presence of systemic inflammation as well as immunosuppressive drug treatment of the BD patients might affect gene expression. CT carriers of FCGR3A rs428888 showed a significantly higher mRNA expression of FCGR3A than CC carriers when testing unstimulated PBMCs (P = 0.005) (Fig. 1). Male individuals also showed a higher mRNA expression of FCGR3A than females when testing unstimulated PBMCs (P = 0.042) (Fig. 2).

**Figure 3:**
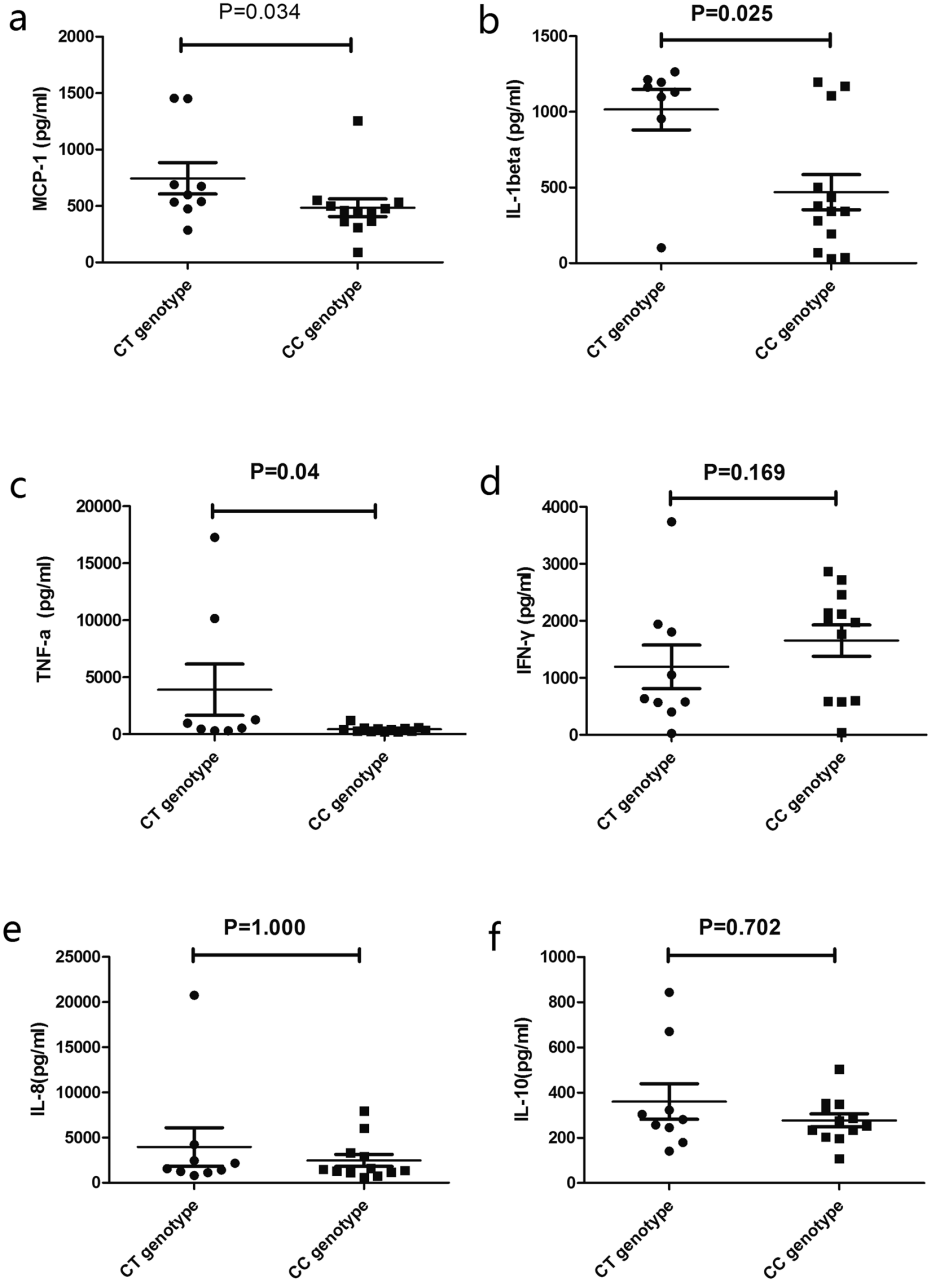
The influence of rs428888 on cytokine production. The production of MCP-1 (**a**), IL-1beta (**b**), TNF-α (**c**), IFN-γ (**d**), IL-8 (**e**), IL-10 (**f**) by LPS stimulated PBMCs from healthy controls carrying different genotypes of rs428888. (male: n = 16 (CT: n = 6; CC: n = 10), female: n = 5 (CT: n = 3; CC: n = 2)).

The effect of different genotypes of rs428888 on cytokine protein production was tested in LPS stimulated PBMCs. We tested the PBMC expression of a number of cytokines, that have been shown to play a role in BD pathogenesis including TNF-α, IFN-γ, IL-1β, IL-8, IL-10 and MCP-1. A higher cytokine protein expression of MCP-1, IL-1β and TNF-α by stimulated PBMCs was found in CT carriers as compared to CC carriers (P = 0.034, P = 0.025, P = 0.04; respectively Fig. 3a–c). The other cytokines tested were not affected by the rs428888 genotype (Fig. 3d–f).

## Discussion

The present study shows that ocular BD in Chinese Han is associated with FCGR3A rs428888. Individuals carrying the CT genotype of FCGR3A rs428888 had a higher risk of developing BD, whereas the CC genotype of this locus provided protection against BD. Further functional studies indicated that mRNA expression of FCGR3A was higher in carriers with the CT genotype of rs428888. Additionally, the protein expression of MCP-1, TNF-α and IL-1β by LPS stimulated PBMCs was significantly increased in carriers with the CT genotype of rs428888 as compared to those carrying the CC genotype.

An earlier study on the association of gene variants of the Fc receptor family in BD patients from a department of Rheumatology, showed a significant association with rs396991 of FCGR3A^26^ Our data showed no significant association between rs396991 of FCGR3A and BD in Chinese Han. This may be due to ethnic differences in BD risk or due the fact that all our patients came from a department of Ophthalmology. A Korean study tested a number of gene variants including FCGR3A in a cohort of 61 BD patients with severe uveitis and also found a significant association (p = 0.047) with FCGR3A/rs396991^27^. The association was weak and the authors did not correct for multiple comparisons.

It has been shown that Fc receptors play a major role in orchestrating cytokine production and maintaining immune homeostasis^28^. Moreover, multiple genetic variations have been identified for the various Fc receptors, in particular the FCGRs, which affect receptor function and were shown to be associated with several autoimmune diseases^16^. A meta-analysis showed that an FCGR3A polymorphism was found to be associated with RA in Europeans but not in Asians^29^. Another family-based study was performed in 119 SLE cases and 316 family members of these cases and the results showed that FCGR3A/rs428888 was involved in genetic susceptibility to SLE in Chinese^30^.

Fc receptor-like genes (FCRLs) which are homologous to the FCGRs in structure, contain six Ig superfamily members that are known as FCRL1–FCRL6 according to their chromosomal order^31^. Previous studies showed that polymorphisms of FCRLs were involved in the development of many autoimmune disorders such as rheumatoid arthritis (RA), autoimmune thyroid disease (AITD) and Graves’ disease^32,33^. Earlier studies from our research group showed a significant association with SNPs of FCRL3 in BD^34^. This gene was not repeated in the current study, but no significant associations were found for the other FCRLs tested.

The FCGR3A gene is located on chromosome 1q23, and interacts with the Fc domain of immunoglobulin G. It plays an important role in the clearance of immune complexes and in antibody dependent cellular cytotoxicity (ADCC)^35^. It is shown to be constitutively expressed as a transmembrane protein on NK cells, tissue specific macrophages, monocytes, dendritic cells and subsets of T cells^36^. It was reported that the number of NK cells in aqueous humor and peripheral blood of BD patients increases^37,38^ and NK cells have been considered to play an important role in controlling BD inflammation^39,40^. Whether FCGR3A variants affect NK cell function is not yet known and deserves further study. We performed functional tests using PBMCs and showed that the CT risk genotype of FCGR3A/rs428888 is associated with an increased FCGR3A gene expression. A higher expression of this receptor might play a role in the exaggerated inflammatory response to microbial antigens, which is currently one of the mechanisms thought to cause BD. We additionally showed that healthy carriers of the CT genotype had an increased production of TNF-α, IL-1beta and MCP-1 by LPS stimulated PBMCs. LPS can activate many cells related to inflammation, induce the release of proinflammatory cytokines and may eventually lead to systemic inflammation^41,42^.TNF-α and IL-1beta have been regarded as inflammatory markers and important regulators of Th17 cell differentiation. Th17 cells are defined by their ability to produce IL-17 besides other proinflammatory cytokines^43^, which all play important roles in the pathogenesis of BD. Our findings are in agreement with previous findings showing that the MCP-1 level in active BD patients is much higher than in normal controls^44^.

Our study has a number of limitations. First of all, the FCGR3A gene expression was only examined in healthy genotyped individuals, since our patients show a variable degree of systemic inflammation and are often under treatment with immunosuppressive agents, which might affect the response of PBMCs. Further functional experiments concerning FCGR3A expression in genotyped BD patients with varying degrees of disease severity are required to address this subject. We tested PBMCs and future functional analysis using isolated cell populations may shed more light on which cell types are especially relevant concerning Fc receptor genes expression. It should be noted that most of our patients are male and that the control group was not exactly matched for gender. As yet we did observe an effect of gender on FCGR3A expression. As mentioned above, all our patients had uveitis and came from a department of ophthalmology and further multidisciplinary study should be performed to investigate whether the observed association is dependent on possible subtypes of BD. It should also be noted that we tested all currently known SNPs as reported in earlier studies concerning the association of FcR family genes with autoimmune or autoinflammatory diseases and it is possible that we might have missed unknown SNPs. Furthermore, only common variants were examined and the potential correlation between rare genetic variants and BD will necessitate detailed sequence analysis.

In conclusion, our results show that FCGR3A/rs428888 confers genetic susceptibility for ocular BD in Chinese Han. The risk genotype of FCGR3A/rs428888 regulates FCGR3A expression and production of the proinflammatory cytokines IL-1β, TNF-α and MCP-1.

## Materials and Methods

### Case-control cohorts

In the present study, all participants including 1022 BD cases and 1803 controls came from the Department of Ophthalmology of the First Affiliated Hospital of Chongqing Medical University and were recruited between April 2008 to October 2015. BD cases were diagnosed in accordance with the International Study Group criteria^45^. In parallel, random control groups were matched geographically and ethnically with the cases. A two stage case–control study was performed. There were 449 BD cases and 658 normal controls in the first stage. For the validation of data obtained in the first study we used a different set of 573 cases and 1145 controls for the second stage. Written informed consent was obtained from all participants and the study was approved by the First Affiliated Hospital of Chongqing Medical University Clinical Ethics Research Committee. All experimental procedures were conducted in line with guidelines and regulations, and abided by the tenets of the Declaration of Helsinki.

### Single nucleotide polymorphisms (SNPs) selection

SNPs were selected on the basis of previously published reports concerning the association between FcR gene polymorphisms with autoimmune or autoinflammatory disorders^16,21–30^. Haploview 4.2 software was used to analyze Minor Allele Frequency (MAF) and Linkage disequilibrium (LD) (the MAF was required to be greater than 0.05 in Chinese Han Beijing data, and an r^2^ threshold of 0.8 in LD). By following these above principles, we chose twenty-three SNPs of FC receptor family genes including FCGR2A/(rs1801274, rs6658353, rs10800309), FCGR2B/(rs1050501, rs10917661, rs12118043, rs1249347), FCGR3A/(rs396991, rs10919543, rs403016, rs428888, rs486062), FCRL1/(rs4971154), FCRL4/(rs2777963, rs10489674, rs14335), FCRL5/(rs6427384, rs12036228, rs6679793, rs2012199, rs3811035, rs6692977), FCRLB/ (rs4657093). There are no data concerning rs428888 in a Chinese Han Beijing population in the HapMap database and 1000genomes. FCGR3A/rs428888 was shown to be associated with SLE in Chinese Han^30^. Since there were no literature reports concerning the association of autoinflammatory disorders with gene polymorphisms of FCDR, FCER and FCMR, the study did not cover these genes. SNPs of FCRL3 were also not included in this study because these data have been reported previously by our group^32^.

### SNP genotyping

Genomic DNA from peripheral blood leukocytes of all the participants was isolated with the QIAamp DNA Blood Mini Kit (QIAGEN Valencia,CA,USA) according to the manufacturer’s instructions. In the first phase, the majority of SNPs were genotyped using the MassARRAY system platform (Sequenom Inc, CA, USA) and iPLEX Gold Assay. Only rs6658353 was genotyped by TaqMan® SNP Genotyping Assay in the Real-Time PCR system (Applied Biosystems, USA). The genotyping results were respectively analyzed using TYPER Software and TaqMan Genotyper Software. For the second phase study, genotyping were performed by PCR-restriction fragment length polymorphism (PCR-RFLP) method.

### Real-time PCR

Peripheral blood mononuclear cells (PBMCs) of healthy controls were separated by Ficoll-Hypaque density-gradient centrifugation, and cultured with lipopolysaccharide (LPS,100ng/ml, Sigma, Missouri, USA) for 24 hours at a density of 1 × 10^6^ cells/ml. The whole RNA was extracted from LPS-stimulated and non-stimulated PBMCs using TRIzol reagent (Invitrogen, San Diego, California, USA) and the Prime Script RT reagent Kit (TaKaRa, Dalian, China) was used for reverse transcription. FCGR3A gene mRNA expression(primer:5′-GCAGCTAGAAGTCCATATCGG-3′ and 5′-CTTCCTGCCTTTGCCATTCTG-3′) and β-actin gene expression(primer: forward 5′-GGATGCAGAAGGAGATCACTG-3′and reverse5′-CGATCCACACGGAGTACTTG-3′) were conducted on an ABI 7500 real-time system. Relative gene expression levels were measured using the 2^−ΔΔCt^ method after data were normalized to mRNA β-actin.

### Enzyme-linked immunosorbent assay (ELISA)

The concentration of TNF-α, IL-8, IFN-γ, IL-10, IL-1β and MCP-1 in culture supernatants of PBMCs were examined using human Duoset ELISA development kits (R&D Systems, Minneapolis, USA).

### Statistical analysis

Hardy-Weinberg equilibrium (HWE) was measured through the SHEsis website. Differences of genotype and allele frequencies between cases and controls were analyzed using the χ2 test with the SPSS 17.0 software. The p values were corrected to Pc with the method of Bonferroni for correction of multiple comparisons, and it was considered to be significant when Pc was less than 0.05. FCGR3A expression levels and various cytokines between the two genotype groups were analyzed by the independent samples T-test or nonparametric Mann-Whitney U test.

## Electronic supplementary material

Supplementary information
