# Supplementary material for: Analysis of the association between Fc receptor family gene polymorphisms and ocular Behçet’s disease in Han Chinese
Source: Sci Rep. 2018 Mar 19;8:4850. doi: 10.1038/s41598-018-23222-8 (PMC5859267; doi:10.1038/s41598-018-23222-8)
Supplement: Supplementary file 1 — Supplementary information [file 41598_2018_23222_MOESM1_ESM.pdf]

Analysis of the association between Fc receptor family gene polymorphisms and ocular Behçet's disease in Han Chinese

Donglei Zhang [1,2], Jieying Qin[1], Lin Li[1], Guannan Su[1], Guo Huang[1], Qingfeng Cao[1], Aize Kijlstra[3], Peizeng Yang[1]

1 The First Affiliated Hospital of Chongqing Medical University, Chongqing Key Laboratory of Ophthalmology and Chongqing Eye Institute, Chongqing, P. R. China.

2 First Hospital of Shanxi Medical University, Taiyuan, Shanxi, P. R. China.

3 University Eye Clinic Maastricht, Maastricht, The Netherlands.

Correspondence to: Professor Peizeng Yang, MD, Ph.D.,

Department of Ophthalmology

The First Affiliated Hospital of Chongqing Medical University

Chongqing Key Laboratory of Ophthalmology and Chongqing Eye Institute

1 You Yi Road, Yu Zhong District

Chongqing, 400016 China

Phone: +8623 89012851;

FAX: +8623 89012851;

Email: peizengycmu@126.com

Table S1 Polymorphisms of other Fc receptor genes in ocular BD

Gene	SNP	Allele / Genotype	BD N	BD %	Control N	Control %	PforBD	Pc	OR（95%CI）
FCGR2A	rs1801274	AA	191	44.3	288	43.5	0.808	NS	1.031（0.807-1.316）
		AG	192	44.5	299	45.2	0.824	NS	0.973（0.762-1.241）
		GG	48	11.1	74	11.2	0.976	NS	0.994（0.676-1.462）
		A	574	66.6	875	66.2	0.846	NS	1.018（0.849-1.221）
	rs10800309	AA	229	51	366	51.7	0.818	NS	0.973（0.768-1.232）
		AG	185	41.2	282	39.8	0.643	NS	1.059（0.832-1.347）
		GG	35	7.8	60	8.5	0.682	NS	0.913（0.591-1.410）
		A	643	71.6	1014	71.6	0.997	NS	1.000（0.830-1.203）
	rs6658353	CC	26	7.3	81	13.9	0.002	NS	0.483（0.304-0.768）
		CG	144	40.2	216	37.1	0.351	NS	1.137（0.868-1.490）
		GG	188	52.5	284	48.9	0.28	NS	1.157（0.889-1.505）
		C	196	27.4	378	29.9	0.018	NS	0.782（0.637-0.960）
FCGR2B	rs1249347	CC	399	88.9	637	89.2	0.852	NS	0.965（0.661-1.407）
		CG	48	10.7	74	10.4	0.86	NS	1.035（0.705-1.520）
		GG	2	0.4	3	0.4	0.949	NS	1.060（0.176-6.371）
		C	846	94.2	1348	94.4	0.848	NS	0.966（0.674-1.383）
	rs1050501	TT	399	89.3	637	89.6	0.852	NS	0.965（0.661-1.407）
		CT	48	10.7	74	10.4	0.86	NS	1.035（0.705-1.520）
		T	846	94.6	1348	94.7	0.848	NS	0.966（0.674-1.383）
	rs10917661	CC	341	85	591	84.8	0.913	NS	1.019(0.723-1.437)
		CT	60	15	103	14.8	0.934	NS	1.015(0.719-1.433)
		TT	-	-	3	0.4	-	-	-
		C	742	92.5	1285	92.1	0.775	NS	1.049(0.756-1.456)
	rs12118043	AA	-	-	2	0.3	-	-	-
		AC	76	18.9	119	19.9	0.669	NS	0.933(0.677-1.285)
		CC	326	81.1	476	79.7	0.596	NS	1.090(0.792-1.501)
		C	728	90.5	1071	89.7	0.534	NS	1.100(0.814-1.487)
FCGR3A	rs486062	TT	219	50.8	300	45.4	0.079	NS	1.243（0.975-1.585）
		CT	212	49.1	361	54.6	0.079	NS	0.804（0.631-1.026）
		T	650	75.4	961	72.7	0.159	NS	1.152（0.946-1.402）
	rs396991	AA	236	52.6	326	45.6	0.021	NS	1.322（1.044-1.675）
		CA	181	40.3	309	43.2	0.328	NS	0.887（0.698-1.128）
		CC	32	7.1	80	11.2	0.022	NS	0.609（0.397-0.935）
		A	653	72.7	961	67.2	0.0049	NS	1.301（1.082-1.563）
	rs403016	CC	338	81.6	516	84.7	0.192	NS	0.802(0.575-1.118)
		CG	76	18.4	93	16.3	0.192	NS	1.248(0.895-1.740)
		C	752	90.8	1125	92.4	0.213	NS	0.818(0.596-1.123)
	10919543	AA	256	63	405	66	0.324	NS	0.877(0.675-1.139)
		AG	134	33	178	29	0.179	NS	1.204(0.919-1.578)
		GG	16	3.9	30	4.8	0.473	NS	0.797(0.429-1.482)

		A	646	79.6	978	79.8	0.63	NS	0.947(0.759-1.182)
FCRL1	rs4971154	CC	80	18.7	111	16.9	0.454	NS	1.129（0.822-1.550）
		CT	215	50.2	322	49	0.712	NS	1.047（0.821-1.336）
		TT	133	31.1	223	34	0.317	NS	0.875（0.674-1.136）
		C	375	43.8	544	41.5	0.28	NS	1.101（0.925-1.310）
FCRL4	rs2777963	AA	369	85.6	556	84	0.466	NS	1.135（0.808-1.594）
		AG	58	13.5	103	15.6	0.338	NS	0.844（0.596-1.194）
		GG	4	0.9	3	0.4	0.336	NS	2.058（0.458-9.0240）
		A	796	92.3	1215	91.7	0.628	NS	1.082（0.787-1.488）
	rs14335	CC	67	14.9	112	15.6	0.74	NS	0.946（0.681-1.314）
		CT	203	45.2	334	46.6	0.632	NS	0.944（0.745-1.196）
		TT	179	40	270	37.7	0.462	NS	1.095（0.860-1.395）
		C	337	37.5	558	38.9	0.487	NS	0.941（0.792-1.117）
	rs10489674	AA	22	4.9	40	5.6	0.607	NS	0.869（0.510-1.483）
		AG	154	34.3	227	31.7	0.367	NS	1.122（0.874-1.442）
		GG	273	60.8	448	62.7	0.526	NS	0.924（0.725-1.178）
		A	198	22	307	21.5	0.741	NS	1.035（0.845-1.266）
FCRL5	rs6427384	CC	24	5.3	39	5.4	0.94	NS	0.980（0.581-1.653）
		CT	162	36.1	240	33.5	0.371	NS	1.120（0.874-1.434）
		TT	263	58.6	437	61	0.404	NS	0.903（0.710-1.148）
		C	210	23.4	318	22.2	0.508	NS	1.069（0.877-1.304）
	rs6679793	AA	8	1.8	16	2.2	0.596	NS	0.794（0.337-1.870）
		AG	92	20.5	140	19.6	0.697	NS	1.060（0.790-1.423）
		GG	349	77.7	560	78.2	0.846	NS	0.972（0.732-1.292）
		A	108	12	172	12	0.991	NS	1.001（0.715-1.294）
	rs6692977	CC	377	84	577	81	0.38	NS	1.152（0.839-1.582）
		CT	72	16	127	17.8	0.38	NS	0.868（0.632-1.191）
		TT	-		8	1.1	-	-	-
		C	826	92	1281	90	0.101	NS	1.281（0.952-1.723）
	rs3811035	AA	188	43.6	267	40.4	0.3	NS	1.139（0.891-1.456）
		AG	189	43.9	305	46.2	0.444	NS	0.909（0.712-1.160）
		GG	54	12.5	88	13.3	0.7	NS	0.931（0.648-1.338）
		A	565	65.5	839	63.6	0.344	NS	1.091（0.911-1.305）
	rs2012199	CC	2	0.4	8	1	0.226	NS	0.392(0.084-1.871)
		CT	112	23.6	144	19	0.056	NS	1.313(0.993-1.735)
		TT	360	75.9	603	79.9	0.104	NS	0.796(0.604-1.049)
		C	116	12.2	160	10.6	0.21	NS	1.176(0.912-1.517)
	rs12036228	CC	405	85.4	632	83.5	0.359	NS	1.161(0.844-1.598)
		CT	69	14.6	120	15.9	0.54	NS	0.904(0.656-1.247)
		TT	-	-	5	0.6	-	-	-
		C	879	92.7	1384	91.4	0.368	NS	1.151(0.847-1.562)
FCRLB	rs4657093	CC	15	3.2	19	2.6	0.53	NS	1.246(0.627-2.477)
		CT	131	27.6	209	28.1	0.857	NS	0.977(0.755-1.263)

		TT	329	69.3	517	70	0.961	NS	0.994(0.774-1.275)
		C	161	16.9	247	16.6	0.811	NS	1.027(0.826-1.277)

SNP, single nucleotide polymorphism ; Pc, Bonferroni corrected p value; NS, not significant; OR, odds ratio; 95 % CI, 95 % confidence interval.

Table S2. Linkage disequilibrium(LD) between the five snps of FCGR3A using our data.

	rs10919543	rs396991	rs403016	rs428888	rs486062
rs10919543	NA	0.3271003	1.01E-04	0.000723	3.69E-06
rs396991	NA	NA	6.94E-05	0.000526	1.25E-06
rs403016	NA	NA	NA	0.709478	1.43E-03
rs428888	NA	NA	NA	NA	1.45E-03
rs486062	NA	NA	NA	NA	NA

Numbers indicate correlation coefficient (r^2) values

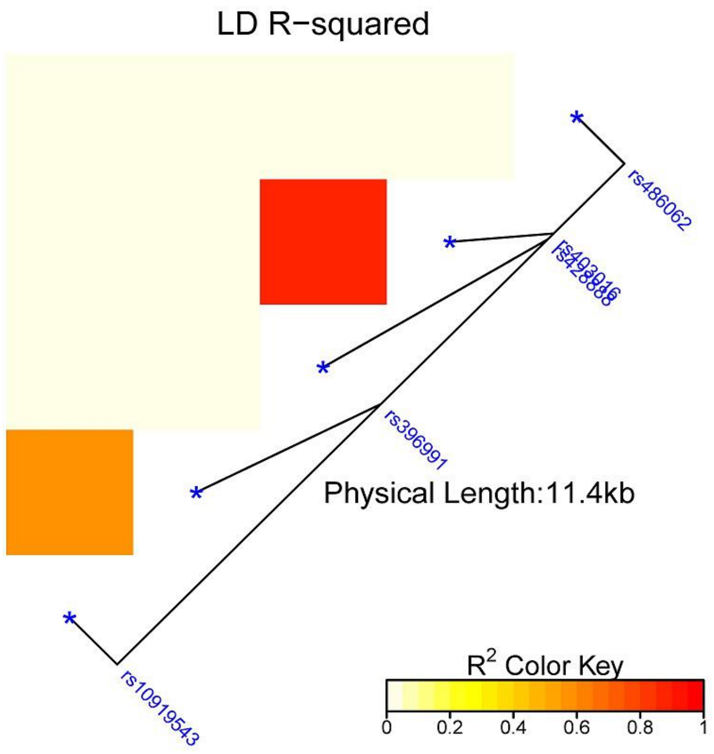

FigureS1. Heatmap illustrate linkage disequilibrium (LD) between the five SNPs of FCGR3A using our own data.
